# A Trace Mineral Injection before Joining and Lambing Increases Marking Percentages and Lamb Weights on Diverse Farms in Victoria, Australia

**DOI:** 10.3390/ani13010178

**Published:** 2023-01-03

**Authors:** Paula A. Gonzalez-Rivas, Graham R. Lean, Michael Chambers, Jerry Liu

**Affiliations:** 1 Virbac Australia Pty Ltd., 361 Horsley Road, Milperra, NSW 2214, Australia; 2 School of Agriculture and Food, Faculty of Veterinary and Agricultural Sciences, The University of Melbourne, Melbourne, VIC 3010, Australia; 3 Agrivet Business Consulting, P.O. Box 105, Hamilton, VIC 3300, Australia; 4 Invetus Pty Ltd., Armidale, NSW 2350, Australia

**Keywords:** sheep, injectable trace minerals, trace minerals, joining, marking, lamb survival, immunity, fertility, antioxidants, oxidative stress

## Abstract

**Simple Summary:**

Optimum trace mineral (TM) status in ruminants is essential for fertility and the survival of the offspring by preventing oxidative stress and improving immunity. The benefits of injectable TM (ITM) supplementation in the lead-up to high-demand periods, such as pregnancy and calving, have been demonstrated in cattle. However, scientific evidence of its benefits for sheep fertility and health is scarce. The objective of this field study was to evaluate the effect of an ITM supplement containing Cu, Se, Zn and Mn pre-joining and pre-lambing to ewes on conception rates, ewe and lamb survival and farm profitability. This study demonstrated that supplementing ewes using ITM before joining and lambing can improve lamb survival, weight at weaning and farm profitability. These results help to understand TM supplementation for animal health, performance and farm profitability beyond the treatment of clinical deficiencies.

**Abstract:**

This study was conducted on five commercial farms across Victoria, Australia, between September 2018 and November 2019, where the TM status of ewes was within normal ranges before joining. Mix breed ewes (n = 1484) were randomly allocated to receive either nil treatment (Control) or two injections of an ITM product containing zinc (40 mg/mL), manganese (10 mg/mL), selenium (3 mg/mL), and copper (10 mg/mL); 0.2 mL per 10 kg BW (Multimin^®^ plus Copper for Sheep, Virbac (Australia) Pty Ltd., Milperra, NSW, Australia) 30 days before the start of joining and 30 days before the start of lambing. Approximately 90 days after joining, pregnancy status and conception rate were determined by ultrasound. The marking rate was determined approximately four weeks after the end of lambing, and lamb weights were determined at weaning (12 weeks after the end of lambing). In all farms, ITM treatment did not affect the conception rate. The average conception rate was 156 ± 11.0% (*p* > 0.05). The marking rate of ITM ewes was 9% higher than control ewes (95% Confidence Interval 3–21%). Lambs born to ITM ewes were 2.31 kg heavier at weaning than lambs born to control ewes (*p* < 0.001). Although not significant, ewe mortality across farms was 1.3% lower in the ITM group than in the control group. On average, ewes treated with ITM pre-joining and pre-lambing produced more and heavier lambs that represent an extra AU$ 2338 per 100 ewes net benefit for the producer. These results help to understand strategic TM supplementation for animal health, performance and farm profitability beyond the treatment of clinical deficiencies.

## 1. Introduction

Neonatal lamb mortality is a significant animal welfare issue. It represents essential production inefficiency to the sheep industry, while lamb survival directly represents the overall reproductive performance of the flock [1]. Most of lamb mortality occurs in the first 48 h after birth [2]. According to Meat & Livestock Australia (MLA) and other authors, in extensively grazed flocks around half of all losses are related to parturition, including stillbirth (21%), birth injury (18%), and dystocia (9%), followed by starvation-mismothering (25%), death in utero/prematurity (10%), predation (7%), and cold exposure (5%) [3,4,5,6]. In another study, MLA reported a mean cumulative ewe mortality of 2.0% in 2020 and 2.5% in 2019, being the most common causes of mortality septicaemia, primary dystocia and trauma [7]. Meat & Livestock Australia suggests that reducing lamb and ewe losses would significantly improve the profitability, animal welfare and social benefits of sheep enterprises.

Trace minerals (TM) copper (Cu), selenium (Se), zinc (Zn) and manganese (Mn) are essential nutrients required for normal metabolic processes in livestock; they are components of metalloenzymes involved in intracellular reactive oxygen species (ROS) and free radical detoxification and stabilisation of secondary molecules. Superoxide dismutase (SOD) enzymes are Mn, Cu and Zn dependent and are the first line of antioxidant defence, converting superoxide anion (O_2_^−^) to hydrogen peroxide (H_2_O_2_). Selenium mediates the activity of Se-containing antioxidant enzymes, including glutathione peroxidase (GSHPx), which regulates oxidative processes and cell membrane protection. These selenoenzymes catalyse the reduction of H_2_O_2_ and lipid peroxides to their corresponding alcohols and water with reduced glutathione (GSH) as the electron donor [8,9].

Optimum equilibrium between pro and antioxidant molecules before and during gestation is an essential requisite for fertility, placental development, and immune function to improve embryo survival and lambing outcomes by ensuring the survival of the newborn [8,10,11,12,13].

Sub-optimal or marginal levels of Cu, Se, Zn and, or Mn before joining and during pregnancy in breeding females are associated with delayed or depressed oestrus, early embryonic death, and retained placenta [14,15,16] inadequate transfer of TM to the foetus through the placenta, causing impaired foetal growth and metabolism, abnormalities in the development of the central nervous system and the skeleton, and impaired immunity and performance in early life [8,17,18].

Trace minerals are present in low concentrations in the body, and their requirements are not static through the production cycle. Increased demands for TM are associated with physiological and environmental stressors and reproductive and immune function [8]. These extra demands require increased TM intake and changes in homeostatic mechanism (increased absorption or reduced excretion) or the mobilisation of TM from maternal pools to the growing foetus in pregnant animals.

The standard practices of supplementing TM to ruminants include free-choice mineral mixes as loose or block forms or associated with energy and protein supplements. However, intake and utilisation of these supplements are highly variable and depend on animal requirements, voluntary feed intake, soil, forage and water quality and availability, palatability, accessibility, source and physical form of the mineral supplement, and presence of TM antagonists [19,20].

Other forms of TM supplementation include injectable formulations. While some only include one trace element, such as Se (barium selenate, sodium selenate), or Cu (copper glycinate); others provide a water-soluble combination of rapidly absorbed trace minerals containing EDTA-bound Cu, Zn and Mn, and sodium selenite. This injectable trace mineral formulation (ITM) allows livestock producers to deliver specific amounts of a combination of TM to individual animals targeting high-demand periods without antagonist interactions within the gastrointestinal tract [20,21,22,23,24].

The benefits of ITM supplementation in the lead-up to high-demand periods, such as pregnancy and calving, have been demonstrated in cattle [11,25,26,27,28] however, scientific evidence of its benefits for sheep fertility and health is scarce. We hypothesised that strategically timed ITM supplementation to ewes would increase conception rates, and lamb growth rates in addition to ewe and lamb survival and thus represent net economic benefits for producers and improved animal welfare outcomes.

## 2. Materials and Methods

This study was conducted with the approval of the Virbac Animal Ethics Committee (AEC 601/18) in accordance with the Australian Code for the Care and Use of Animals for Scientific Purposes [29].

The study was undertaken in five commercial farms (A, B, C, D, E) across Victoria, Australia, between September 2018 and November 2019.

### Animals

Pasture-fed mixed-breed ewes (n = 1484; 300 ewes per farm approximately) were randomly allocated to receive either nil treatment (Control) or two injections of an ITM product containing zinc (as disodium zinc EDTA 40 mg/mL), manganese (as disodium manganese EDTA 10 mg/mL), selenium (as sodium selenite 3 mg/mL), and copper (as disodium copper EDTA 10 mg/mL) at a dose of 0.2 mL per 10 kg BW (Multimin^®^ plus Copper for Sheep, Virbac (Australia) Pty Ltd., Milperra, NSW, Australia) 30 days before the start of joining and 30 days before the start of lambing.

Thirty days before the pre-joining treatment, ewes were randomly allocated to the treatment groups based on body weight and body condition score. Ewes were identified with coloured ear tags for each treatment.

In three randomly selected farms (A, C and D) blood samples (10 mL) were collected via jugular venipuncture from 10 randomly selected ewes from each treatment group to determine basal levels of glutathione peroxidase (GSHPx; an antioxidant enzyme correlated with Se levels in blood [8]) and copper. This allows to determine whether animals were considered deficient, marginal or adequate in trace minerals and to eliminate the risk of toxicity before the first injection of TM. Blood samples were analysed by a commercial laboratory (Regional Laboratory Services, Benalla, VIC, Australia).

Records of clinical TM deficiencies or toxicities were reviewed for all farms. All farms were not deficient in TM by the absence of clinical TM deficiencies such as white muscle disease, spontaneous fractures, enzootic neonatal ataxia (swayback), or discoloured wool in the last two years. In all farms, there were no risk factors for Cu toxicity, such as the presence of hepatotoxic plants or previous records of Cu toxicity cases.

Approximately 90 days after joining, pregnancy status (pregnant or empty) and conception rate (foetuses per 100 ewes) were determined by ultrasound. Ewes from each treatment group were managed in different paddocks of similar pastures with additional supplementary feed according to their pregnancy status (singles or multiples) as per standard practices for each farm. Lambing ewes were supervised twice a day, and weekly supervision was conducted all other times. The marking rate was determined approximately four weeks after the end of lambing, and lamb weights were determined at weaning (12 weeks after the end of lambing).

Due to data recording issues, data for lamb marking and lamb weaning weights were unavailable for farms E and D, respectively. Weaning weight data were compared using parametric ANOVA (both within and across all farms in Tibco SPOTFIRE S+ V 8.2, 2010. Fixed effects were treatment and farm, and random effects were paddock and sheep ID. Conception, marking, weaning rates, and ewe mortality were compared (within and across farms) using MedCalcs ‘Comparison of Two Rates’ calculator (2021 MedCalc Software Ltd.) which provided the calculated rate, a (Poisson) 95% Confidence Interval, a point estimate of the difference between the two rates and a 95% Confidence Interval around the point estimate. The *p*-value was obtained from a calculated Chi-squared test. Significance was defined as *p* < 0.05.

A partial budget was constructed to assess the net benefit of administering an ITM formulation pre-joining and pre-lambing on lamb survival and weaning weight based on the results of this study. Commodity data was sourced from MLA averaged for the two years up to April 2021. Store lamb prices were based on pricing quoted by AuctionsPlus https://auctionsplus.com.au for crossbred lambs in April 2021, which would apply to lambs at weaning or soon after. Full retail pricing for ITM was supplied by Virbac (Australia) Pty Ltd. Labour was based on current ruling rate for contract farm labour. Values are expressed in AUD.

## 3. Results

In farms A, C, and D, average glutathione peroxidase (GSHPx) and copper status in ewes were within normal ranges; however, some ewes presented marginal levels before joining (Table 1).

In all farms, ITM treatment did not affect the conception rate. The average conception rate was 156 ± 11.0% (*p* > 0.05). The marking rate of ITM ewes was 9% higher than control ewes (95% Confidence Interval 3–21%, 128% ITM vs. 119% control ewes). Lambs born to ITM ewes were heavier at weaning than lambs born to control ewes (2.31 kg; *p* < 0.001). Overall, there was between 0.75 and 4.27 kg benefit to weaning weight with ITM (Table 2). Although not significant, ewe mortality across farms was lower in the ITM group (1.17%) than in the control group (2.49%; *p* > 0.05).

Economic analysis was conducted based on the average data (Table 2). For 100 ewes treated, producing 9 extra lambs at marking, each lamb having 2.3 kg additional at weaning (assuming a 46% dressed percentage and AU$ 8.00 CWT) and reducing the cost associated to labour and ITM treatment, the net benefits of including ITM pre-joining and pre-lambing can be reflected in AU$ 2338 ($23/ewe) for the producer (Table 3).

## 4. Discussion

The field trial results demonstrated that supplementing TM in ewes with a commercial ITM formulation containing Cu, Se, Zn and Mn before joining and lambing can improve lamb survival, weight, and farm profitability. Although not significant, differences in ewe survival between treatments were also observed, potentially adding AU$ 455 extra per 100 treated ewes profit (based on 1.3% survival and AU$ 350/head) and significantly improving welfare outcomes.

These benefits can be a consequence of the role of TM on immunity and health by reducing oxidative stress and enhancing innate and acquired immunity in ewes and lambs [8,9,10]. Differences in management, animal husbandry and general nutrition cannot be negated and might explain differences among farms. Importantly, benefits were observed in farms without clinical TM deficiencies. Therefore, these results help to understand TM supplementation for animal health and performance beyond treating deficiencies.

### 4.1. Trace Minerals and Fertility

Previous studies have demonstrated that pre-joining supplementation with a similar ITM formulation containing Cu, Se, Zn and Mn increased pregnancy rates following a fixed-time artificial insemination protocol in Bos taurus cows and heifers [30], and following embryo transfer in non-deficient Bos indicus × Bos taurus heifers [26]. Similarly, Vedovatto et al. [31,32] reported that a single ITM injection 30 d before AI tended to improve pregnancy rates in Nellore cows with BCS < 5.

Gabryszuk & Klewiec [33] gave non-Se deficient 2- and 3-year-old ewes injections of sodium selenate four weeks before mating (5 mg Se/ewe) and four weeks before lambing (5 mg Se/ewe), similar to the Se doses used in the current protocol and observed increased detection of oestrus (100 vs. 76%) and fertility (100 vs. 68%) in 3-year-old treated ewes compared with control animals, but no effect in younger ewes. Other authors have shown variable or no impact of ITM on pregnancy rates in ruminants [34,35,36]. In the current study, ITM treatment did not affect conception rates in ewes at scanning. The inconsistent results found in the literature can be explained by factors such as breed, animal class, general nutrition, reproductive management, environmental factors, body condition score, TM status at the time of ITM administration and experiment design. In the present study, TM status at the start of the study was not determined in all farms, however some farms were considered marginal-deficient, and pasture analysis was not conducted. More detailed feed analysis is recommended in future experiments.

### 4.2. Trace Minerals during Pregnancy and Their Effects on Early Post-Natal Life

The current study demonstrated that ITM pre-joining and pre-lambing in ewes can improve lamb survival and weight. These benefits can be attributed to the role of TM on immunity and health by reducing oxidative stress and enhancing the lamb’s and ewe’s innate and acquired immunity.

During pregnancy, all tissues, including the placenta and foetus, require large amounts of oxygen due to increased energy demands for growth and development. Reactive oxygen species (ROS) are produced by both the mother and foetus, as part of the normal metabolism. The adequate concentration of ROS is essential for embryo development, implantation, growth and maturation of cells and organs, defence against uterine infections, steroidogenesis, maintenance of pregnancy and the initiation of partum. However, excessive ROS production beyond physiological antioxidant defences may lead to oxidative stress (OS) with harmful effects on both the mother and foetus. Due to OS, alteration in cell structure and function results in embryo resorption, placental degeneration with subsequent alteration in maternal-foetal nutrient and oxygen exchanges, delay in foetal growth, pregnancy interruption, stillbirths, and post-partum diseases [37,38,39,40]. Previous research has demonstrated that ewes experience OS during the peripartum period with an increase in serum ROS at birth and until 36 h post-lambing [13,41].

Newborn lambs are also prone to develop free radical-induced damage and OS due to the sharp variations in pO_2_ around parturition. Increased aerobic metabolism after birth will increase ROS resulting in OS if the antioxidant system cannot counteract their effects [42,43]. Furthermore, the impact of prolonged birth and intrapartum asphyxia due to a weak dam, foetal entanglement or malpresentation leads to decreased cellular oxygen and glucose availability, triggering an increase in anaerobic glycolysis and ischemia with a subsequent reperfusion injury which induces production of ROS and OS [44,45]. These phenomena are considered contributing factors to the pathogenesis of many newborn diseases in several mammalian species, such as long-term motor and neurodevelopmental disabilities, thyroid gland dysfunction, and respiratory, digestive, and cardiac syndromes [46,47,48,49].

Injectable trace mineral formulations similar to the one used in this study have been demonstrated to increase the activity of antioxidant enzymes (SOD and GSHPx) in cattle after treatment [31,32,50,51]. Although the antioxidant enzyme responses after treatment were not determined in this study, the authors suggest that the reduced ewe losses and higher marking rates observed in the current trial can be consequences of improvements in the antioxidant status and health of ewes and their lambs.

The implications of optimum maternal TM status and placental transfer on foetal and neonatal ruminant metabolism have been studied for several decades. During gestation, the ruminant foetus is dependent upon its dams for the transfer of TM via the placenta [8,18,52] and additional nutritional inputs are required to support pregnancy and lactation above the requirements for maintenance and growth. The situation is exacerbated if ewes have grazed rapidly growing lush pasture or young crops pre-lambing since the rapid pasture growth in spring may dilute the TM content in the pasture [53]. Several studies [52,54,55,56,57,58] have shown Se, Zn, Cu, and Mn levels in the ovine and bovine conceptus (embryo and associated extraembryonic membranes) and foetal liver are several folds greater than in the surrounding reproductive tissues and maternal liver as gestation advances.

Recent experiments conducted in ruminants have demonstrated that maternal supplementation of TM using oral or injectable formulations during pregnancy modifies metabolism [59], body composition [59,60], TM status [61,62,63], reproductive [57], immune function [57,62,64], and thermoregulation [65] of the progeny during the early post-natal life. Jacometo et al. [64] suggested maternal TM supplementation reduces systemic inflammation in calves after birth and allows additional energy (glucose) to be spared for growth. We, therefore, suggest that supplementing ewes with ITM pre-lambing could have elicited a better post-natal health status and development in lambs [e.g., by enhancing glucose metabolism, energy utilisation, and growth] and sparing glucose from maintenance functions in favour of tissue growth and improved innate immunity, resulting in heavier lambs at weaning.

Cartilage and bone development require Zn, Mn and Cu, as they are cofactors of enzymes that synthesise chondroitin-sulphate and others involved in the cross-linking of protein chains in elastin and collagen of cartilage [66,67]. Whether improved bone growth and density account for increasing body weight in lambs from ewes supplemented with ITM pre-joining and pre-lambing in this trial remains to be determined and ensures further investigation.

Maternal supplementation with organic Cu, Mn, and Zn during late gestation has been demonstrated to improve colostrum quality by increasing the concentration of immunoglobulins and reducing calf mortality, improving calf serum antioxidant capacity and immunoglobulin concentrations compared with calves from non-supplemented cows [68,69]. Direct effects of ITM on maternal TM status and indirect effects through passive maternal transfer (including colostrum TM and IgG) in the current trial should be considered but could not be accurately discerned and ensures further investigation. We hypothesise that ewes supplemented with ITM pre-lambing have improved trace mineral transfer via the placenta and better colostrum quality and absorption in lambs.

Van Niekerk et al. [70] gave ewes a single injection of Cu heptanoate (a long-acting Cu source; 12.5 mg Cu per ewe) at varying times after joining in three consecutive years. Although there was no effect on lamb birth or weaning weights, there was a trend towards improved lamb survival compared with untreated animals (+13%). The authors conclude that improved lamb survival after Cu supplementation constitutes a definite economic benefit under commercial farming conditions. This conclusion agrees with the results obtained in the present research.

Gabryszuk & Klewiec [33] gave non-Se deficient 2- and 3-year-old ewes injections of sodium selenate four weeks before mating (5 mg Se/ewe) and four weeks before lambing (5 mg Se/ewe). There was increased lamb body weight at day 28 (10.9 vs. 9.4 kg) and lamb ADG in the first 28 days (241.8 vs. 197.7 g) in 3-year-old ewes compared with non-treated ewes. Administration of Se also increased ADG in the first 28 days (220.1 g/day vs. 186.1 g/day) in lambs of 2-year-old ewes compared with controls, but lambs born to younger ewes had lower live weight at birth (3.9 kg versus 4.3 kg in control). The authors attributed the better responses observed in 3-year-old ewes to the fact that after pregnancy and lactation, older ewes could not replenish the lost stores of Se before entering the subsequent pregnancy. Supplementation of ewes during pregnancy with Se boosts the Se status of lambs at birth by increasing their liver reserves and the transfer of Se in colostrum/milk, minimising the risk of nutritional myopathies such as white muscle disease [8].

Selenium is essential for thyroid hormone function as a cofactor in iodothyronine deiodinase, responsible for the conversion of thyroxine (T4) to the more metabolically active 3,3′-5 triiodothyronine (T3) [71]. Selenium deficiency is suggested to induce notable changes in circulating amounts of thyroid hormones in lambs, which may be associated with post-natal disorders such as stillbirths and neonatal deaths [72]. Zinc is also associated with thyroid hormone activation; it is required by the T3 receptor to adopt its biologically active conformation [73]. Therefore, we suggest that combined Se and Zn supplementation using ITM may contribute to improved thyroid function, thermoregulation, basal metabolism and, consequently, to improved lamb survival and growth in this experiment. Nevertheless, studies investigating the interaction of Se, Zn and I supplementation in livestock are scarce, but the positive outcomes guarantee further investigation [74,75,76].

Mild Zn deprivation in pregnant ewes reduces the number of lambs born, lamb survival, and birth weight [8]. It is associated with prolonged labour, retained placenta and pregnancy toxaemia due to Zn deficiency-induced anorexia in ewes [77]. In this study, we suggest that increasing Zn status can contribute to improved ewe and lamb health.

Responses to TM supplementation in pregnant ruminants are sometimes inconclusive in the literature. Many studies evaluate the effect of single trace mineral supplementation in marginal or deficient animals; published literature varies in the chemical form (inorganic or chelated), the method (in-feed, dosed orally or injected), the inclusion (as per requirements or supra-nutritional) or they have confounding factors across experiments including, but not limited to, trace mineral antagonists, environmental conditions, duration of supplementation, and maternal nutrition status [78,79].

The outcomes of this study suggest that similar to what has been demonstrated in cattle [20,24] the use of a commercial ITM product containing a combination of TM allows sheep producers to supplement specific amounts of TM to individual animals improving fertility, health, performance and farm profitability.

### 4.3. Net Economic Benefits of ITM Supplementation

The partial budget results indicate a high profitability improvement of AU$23/ewe. To this improvement in context, the long-term average Prime Lamb Enterprise Gross Margin since 1970/71 recorded within Agriculture Victoria’s Livestock Farm Monitor Project is AU$36/dry sheep equivalent (dse). This provides producers to optimise the nutritional status of the joined ewe, and by doing so, significantly increase their profitability.

## 5. Conclusions

These results demonstrate that increasing TM status in ewes using a commercial ITM supplement containing Cu, Se, Zn and Mn before joining and lambing can improve lamb survival and weight at marking. Notably, the benefits were observed in farms without signs of TM deficiency. Therefore, these results help to understand TM supplementation for animal health and performance beyond treating clinical deficiencies. Furthermore, we have demonstrated the economic benefits of a strategic TM supplementation in the lead-up to high-demand periods in sheep. Further studies are recommended to determine the effect of ITM treatment pre-joining and pre-lambing on ewes and lambs’ metabolic, immune and antioxidant functions.

## Figures and Tables

**Table 1:** Mean (SD) of blood GSHPx* and Cu levels pre-joining per treatment group.

Item (Reference Range)	Farm A		Fam C		Farm D	
	ITM	Control	ITM	Control	ITM	Control
GSHPx * (50–550 U/gHb)	351.4 (83.0)	350.9 (57.0)	147.2 (33.2)	139.6 (21.9)	110.7 (33.7)	145.7 (43.3)
Cu (9–25 umol/L)	15.8 (2.2)	16.3 (2.4)	10.62 (2.8)	12.6 (0.6)	14.6 (2.2)	13.8 (1.5)

Results are the average of 10 ewes per treatment group. ITM (Injectable Trace Mineral) * Glutathione peroxidase enzyme activity; *p* > 0.05 within farm.

**Table 2:** Differences in marking rates and weaning weights (point estimates of ITM minus Control, 95% Confidence Intervals for the difference and observed *p*-values) for lambs born to ewes treated with injectable trace minerals (ITM) and control ewes.

Farm	A	B	C	D	E	ALL
Marking Rate (%)						
Lower 95% CI	9%	10%	−39%	−18%	NA	3%
Point Estimate of Difference (ITM—Control)	17%	16%	−10%	8%	NA	9%
Upper 95% CI	43%	42%	19%	34%	NA	21%
*p*-value	0.193	0.236	0.503	0.525	NA	0.144
Weaning Weights (kg)						
Lower 95% CI	3.14	−0.30	0.26	NA	0.01	1.43
Point Estimate of Difference (ITM—Control)	4.27	0.75	1.94	NA	0.95	2.31
Upper 95% CI	5.39	1.80	3.62	NA	1.89	3.18
*p*-value	<0.001	0.158	0.024	NA	0.047	<0.001

**Table 3:** Net benefit of using injectable trace minerals pre-joining and pre-lambing in ewes.

For 100 Ewes Treated	Benefit	Value	Total (AUD)
Extra lambs at marking	9%	$175	$1575
Extra kg/lamb at weaning	2.3 kg	128 × 2.3 kg × 46% dressed × $8.00 CWT	$1083
Cost	$1.10/head ITM × 2 + $0.50 labour × 2 = $3.20/ewe	100 × $3.20	$320
Net benefit	Per 1000 ewes		$23,380
	Per 100 ewes		$2338
	Per ewe		$23.38

## Data Availability

Data available upon request.
